# Targeting Non-Oncogene Addiction: Focus on Thyroid Cancer

**DOI:** 10.3390/cancers12010129

**Published:** 2020-01-04

**Authors:** Maria Chiara Anania, Tiziana Di Marco, Mara Mazzoni, Angela Greco

**Affiliations:** Molecular Mechanisms Unit, Department of Research, Fondazione IRCCS Istituto Nazionale dei Tumori, 20133 Milan, Italy; mariachiara.anania@istitutotumori.mi.it (M.C.A.); tiziana.dimarco@istitutotumori.mi.it (T.D.M.); mara.mazzoni@istitutotumori.mi.it (M.M.)

**Keywords:** non-oncogene addiction, thyroid cancer, functional screening, non-oncogenes, tumor vulnerabilities, therapeutic targets

## Abstract

Thyroid carcinoma (TC) is the most common malignancy of endocrine organs with an increasing incidence in industrialized countries. The majority of TC are characterized by a good prognosis, even though cases with aggressive forms not cured by standard therapies are also present. Moreover, target therapies have led to low rates of partial response and prompted the emergence of resistance, indicating that new therapies are needed. In this review, we summarize current literature about the non-oncogene addiction (NOA) concept, which indicates that cancer cells, at variance with normal cells, rely on the activity of genes, usually not mutated or aberrantly expressed, essential for coping with the transformed phenotype. We highlight the potential of non-oncogenes as a point of intervention for cancer therapy in general, and present evidence for new putative non-oncogenes that are essential for TC survival and that may constitute attractive new therapeutic targets.

## 1. Introduction

### Non-Oncogene Addiction (NOA) and Tools for Its Identification in Cancer Cells

It is well established that cancer cells are characterized by several hallmarks that include uncontrolled proliferation and insensitivity to anti-growth signals, evasion of apoptosis, unlimited replicative potential, with acquired capabilities in tissue invasion and metastasis, and sustained angiogenesis [1]. These properties are generally acquired through alterations in oncogenes (gain of function mutation, amplification, overexpression) and tumor suppressor genes (loss-of-function mutation, deletion, epigenetic silencing). Tumor phenotype generally relies on the activity of specific driver alterations of oncogenes and related pathways [2], which to date have been considered the optimal targets for therapy [3]. Indeed, a variety of molecular-targeted agents have been developed such as drugs targeting HER2 (Erb-b2 receptor tyrosine kinase 2) in breast cancer [4], BRAF (v-Raf murine sarcoma viral oncogene homolog B1) in melanoma [5], EGFR (epidermal growth factor receptor) and ALK (anaplastic lymphoma receptor tyrosine kinase) in non-small cell lung cancer [6], KIT (KIT proto-oncogene, receptor tyrosine kinase) in gastrointestinal tumors [7], BCR-ABL (breakpoint cluster region-abelson tyrosine-protein kinase 1) in chronic myeloid leukemia [8], RET (rearranged during transfection) in thyroid and lung cancer [9,10], and TRK (tropomyosin receptor kinase) in a variety of different cancer types [11]. Although successful at the clinic, target therapies present some limitations, such as modest and insufficient response, or the occurrence of resistance. This underlines the need of new strategies of intervention. 

**Figure 1:**
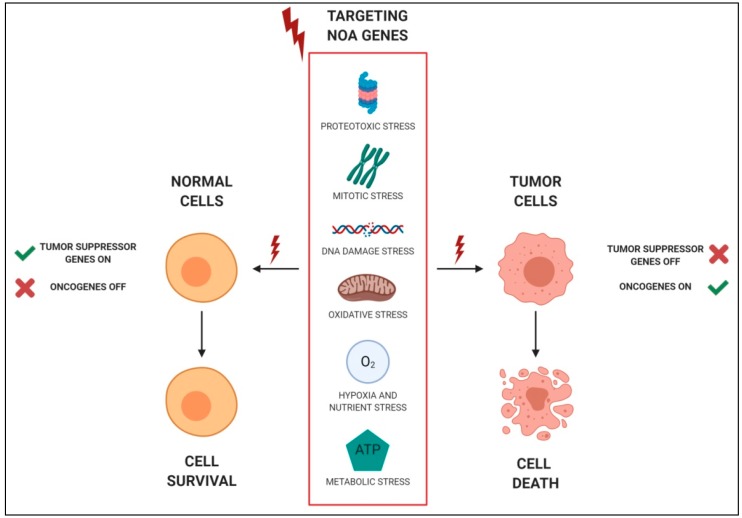
Schematic representations of non-oncogene addictions (NOAs). Examples of stress support pathways to which tumor cells, but not normal cells, are addicted. Interfering with these pathways, which represent NOAs, can be selectively lethal for tumor cells. Figure created with BioRender.

At variance with normal cells, tumor cells are subjected to persistent activation of oncogenic pathways that are responsible for increased levels of cellular stress [12]. To cope with this “stress phenotype”, they are addicted to the activity of a plethora of essential genes, not mutated or aberrantly expressed or responsible for tumor initiation, but necessary to support the transformed phenotype. This phenomenon has been defined “non-oncogene addiction” (NOA) [13]. Being a feature of tumor cells, targeting NOA can represent a point of intervention for cancer therapy, with the concomitant advantage of being ineffective for normal cells. Luo and colleagues attributed NOA genes to several additional hallmarks of cancer that are involved in DNA damage and replication stress, mitotic stress, proteotoxic stress, metabolic stress, and oxidative stress [13] (Figure 1), which are clearly interconnected with each other. 

Hereafter, we will provide some relevant examples of non-oncogenes in cancer cells. Some faithfully reflect the definition, being not mutated or aberrantly expressed in tumors in comparison to the normal counterpart; other dependencies have been expressly considered true NOAs, despite being frequently up-regulated, because, as a compensatory mechanism, they are essential in sustaining the malignant phenotype driven by oncogenes.

At variance with normal cells, upon activation of oncogenes and the high degree of replication, cancer cells have to cope with the increase of genomic instability and aneuploidy, as well as spontaneous DNA damage, which are closely connected with DNA damage stress response (DDR) pathways. Indeed, targeting members of DDR such as ATM (ataxia telangiectasia mutated) and CHK1 (checkpoint kinase 1), amplifies DNA damage and induces lethality on tumor cells [14,15]. Forced replication of tumor cells is consequently linked to mitotic stress, characterized by a huge amount of errors on chromosome segregation. The use of inhibitors of mitotic regulators, such as taxol or inhibitors of PLK1 (polo like kinase 1) and the Aurora-A/B mitotic kinases, has been demonstrated to promote mitotic stress overload on tumor cells [13].

Replication and mitotic stress consequently imply the alteration of protein homeostasis and clearance, leading to the generation of a large burden of misfolded proteins, resulting in endoplasmic reticulum (ER) stress, engulfment of autophagic machinery, and, finally, cell death. Thus, cancer cells are highly dependent on a tightly regulated machinery of protein homeostasis involving the ubiquitin proteasome system, macroautophagy, and unfolded protein response [16]. One of the first examples of NOA is *HSF1* (the heat-shock factor 1) gene, not mutated in cancers, which encodes the transcription factor deputy to orchestrate the heat-shock response upon proteotoxic stress, through the involvement of HSP90 (heat shock protein 90) [12]. Several HSP90 inhibitors have entered clinical studies. HDAC6 (histone deacetylase 6) is a cytosolic class-IIb histone deacetylase, involved in several process such as protein degradation both via aggresomes and regulation of HSP90 chaperone activity [17]. Being essential in coping with accumulation of protein aggregates and damaged mitochondria, HDAC6 has emerged as a clear non-oncogene for inflammatory breast cancer [18]. Its inhibitor ricolinostat (ACY1215) has been demonstrated to selectively kill different types of cancer cells and has entered clinical trials [17].

Metabolic reprogramming represents an advantage for cancer cells [19], with glucose and glutamine metabolism having a central node in sustaining the cancer phenotype. Cancer metabolism and its reprogramming can definitely be considered a reservoir of NOAs to be targeted. Indeed, the inhibition of glycolysis through the administration of non-metabolizable glucose analogues (2-deoxyglucose or 3-bromopyruvate) [20] or inhibition of lactate dehydrogenase (LDH) [21] represent a therapeutic intervention for tumors, which is not harmful to normal cells. The use of metformin, a well-known antihyperglycemic agent that inhibits the PI3K (phosphatidylinositol-3-kinase)/AKT (serine/threonine kinase 1)/mTOR (mammalian target of rapamycin) pathway, has been considered a strategy to mimic glucose deprivation in many tumors [22]. Recent studies have focused on the alteration of amino acid metabolism, as it is known that cancer cells rely on the availability of non-essential amino acids such as glutamine [23]. Some tumors, especially those with *RAS* (rat sarcoma viral oncogene homolog) mutation, are dependent on macropinocytosis for amino acid supply [24], thus representing the best candidates to be treated with vesicle formation inhibitors. Recently, Li et al. found that mitochondrial SIRT3 (sirtuin 3) is required for diffuse large B cell lymphomas (DLBCLs), but not normal germinal center B cells, to regulate glutamine flux to the tricarboxylic acid (TCA) cycle and acetyl-coenzyme A (CoA) pools [25]; this led to the development of the sirtuin inhibitor YC8-02, which is able to preferentially kill DLBCL cells. Tumor cells are also highly dependent on the activity of 3-hydroxy-3-methyl-glutaryl-coenzyme A (HMG-CoA) reductase for the production of cholesterol and mevalonate pathway end-products. Accordingly, HMG-CoA reductase inhibitors (lovastatin, simvastatin, pravastatin, and atorvastatin) are being reconsidered for cancer prevention, treatment, and chemosensitization [26]. mTOR signaling can be considered as the hub of cell metabolism, as it regulates nucleotide synthesis, lipid synthesis, and glucose metabolism [27]. The use of mTOR inhibitors such as everolimus has been approved to treat some human cancers [28]. 

Cancer cells are generally overloaded with reactive oxygen species (ROS) in comparison to normal cells [29]. The source of ROS is essentially forced mitochondrial oxidative phosphorylation, the activity of oncogenes known to induce cell senescence [30] and hypoxic conditions outside cells [29]. High ROS levels are responsible for the accumulation of DNA damage, leading to genomic instability, impairment of mitochondrial functionality, and membrane lipid peroxidation [29]. Recent studies have identified the normally non-essential gene *NUDT1* (nudix hydrolase 1) deputy to tune intracellular oxidative damage by removing oxidized nucleotides, whereas *NRF2* (nuclear factor erythroid 2-related factor 2) was found responsible for the transcription of antioxidant enzymes such as *SODs* (superoxide dismutases) and *GST* (glutathione S-transferase) [20]. Cancer therapeutic approaches can include strategies inhibiting or enhancing ROS production. Inhibition of ROS production can be achieved by handling metabolism, such as pushing glycolysis, down-regulating mitochondrial function, and glutathione synthesis through use of antioxidant compounds [20]. On the other hand, when ROS production is enhanced, for example using dichloroacetate, which inhibits pyruvate dehydrogenase kinase (PDK) and pushes mitochondrial oxidative phosphorylation, cells undergo stress overload and succumb to cell death [13].

Until now, we have considered intrinsic tumor categories in which non-oncogenes fall; however several extrinsic tumor vulnerabilities, such as interaction of tumor cells with stroma, angiogenesis, and immune response, should also be considered and provide other important therapeutic targets. Recently, it has been demonstrated that NOA plays an important role in the progression of cancer-associated inflammation [31]; key genes, such as those belonging to the NF-kB (nuclear factor kappa B) family and VEGF (vascular endothelial growth factor) /VEGFR (vascular endothelial growth factor receptor) axis; immunomodulatory factors, such as chemokines/cytokines (*CCL2* (C-C motif chemokine ligand 2), *IL6* (interleukin 6), *IL10* (interleukin 10); and prostaglandins constitute adaptive essential genes for tumor cells in creating an inflammatory and immunosuppressive milieu. This supports the idea of reconsidering drugs, most of which are already on the market, such as COX-2 (cyclooxygenase-2) inhibitors (NSAID (nonsteroidal anti-inflammatory drug), celecoxib), NF-kB inhibitors, and VEGF inhibitors (bevacizumab), which may be effective in treating tumors [31].

**Figure 2:**
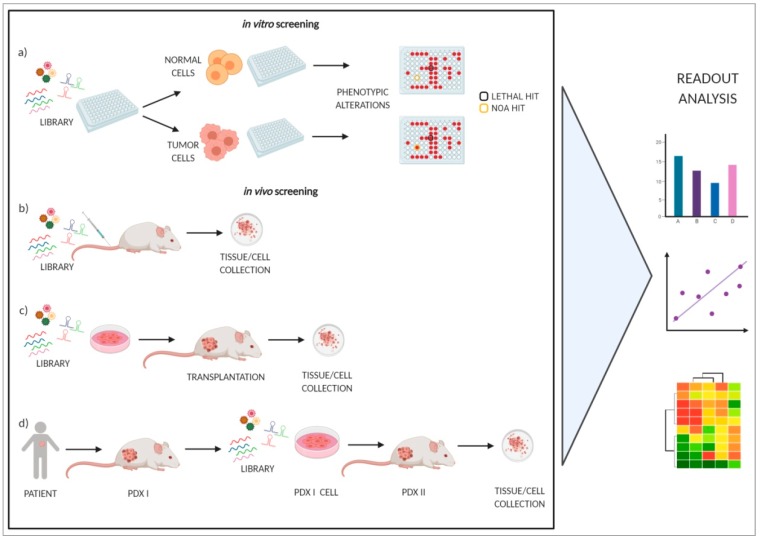
Examples of RNA interference (RNAi) screening approaches. In vitro screening: libraries are directly screened in tumor and normal cells, used as a control **(a)**; in vivo screening: libraries are directly injected in mice (**b**), transfected in cells before transplantation (**c**), or transfected in PDX (patient-derived xenograft)-derived cells (**d**). For all the approaches, cells or tissues were analyzed for phenotypic alterations. Figure created with BioRender.

The traditional approach for cancer target discovery is based on the identification of genetic lesions (mainly oncogenes and tumor suppressors) through the analysis of copy-number variation, transcriptional profiles, epigenetic modifications, and DNA sequence alterations; thus, they are not consequently able to detect NOA genes. The advent of loss-of-function RNA-interference-based genetic screenings, performed both in vitro and in vivo, has overcome this limitation, as they have the potential to unveil a genetic landscape for cancer vulnerabilities, useful in the discovery of new therapeutic targets [13]. A schematic representation is reported in Figure 2.

One of the most cited examples is the discovery of the transcription factor *IRF4* (interferon regulatory factor 4), which is not genetically altered in multiple myeloma cells, but which is essential for growth and survival through an aberrant regulatory network leading to the up-regulation of the oncogene *MYC* [32]. Other in vitro functional screenings allowed for the identification of: genes required for proliferation of mammary tumor cell lines [33,34]; the lethal effect of CHK1 inhibition in neuroblastoma cell lines [35]; synthetic lethal interactions with the KRAS (kirsten rat sarcoma viral oncogene homolog) oncogene in *KRAS*-mutated cancers [36,37,38]; increased mitochondrial dependence upon mTOR addiction of cancer cells [39]; proteasome addiction in basal-like triple negative breast cancers [40]; and *HSPA5* (heat shock protein family A member 5), *NDC80* (NDC80 kinetochore complex component), *NUF2* (NUF2 component of NDC80 kinetochore complex), and *PTN* (pleiotrophin) as vulnerability for ovarian cancer [41]. More recently, large scale studies, aimed to identify common cancer dependencies, have been undertaken [42,43,44,45].

Several in vivo genetic screenings using manipulated cell lines or patient-derived tumors have been performed, which have provided novel insights into mechanisms of tumor growth and maintenance. D’Alesio et al. used a short hairpin RNA (shRNA) lentiviral-based library targeting chromatin modifiers in breast cancer and identified epigenetic vulnerabilities that are not essential for non-transformed mammary epithelial cells [46]. Bossi et al. reported the first in vivo genetic screen of patient-derived metastatic melanoma tumors, unraveling a huge amount and inter-patient heterogeneity of normal genes that are essential for tumor growth [47]. Carugo et al. performed in vivo functional screening of patient-derived pancreatic ductal adenocarcinoma (PDAC) xenografts and attributed a role in protecting PDAC cells from lethal DNA damage accumulation to the WDR5 (WD repeat domain 5)-Myc axis [48]. Rudalska at al. performed a pooled shRNA screening directly in mouse hepatocellular carcinomas, identifying genes whose inhibition increased the therapeutic efficacy of sorafenib [49]. Pooled CRISPR/Cas9 screens have also been used to identify genotype-specific cancer vulnerabilities as potential drug targets. For example, genome-scale pooled CRISPR screening technology was applied to identify vulnerabilities in *RNF43*(ring finger protein-43)-mutant pancreatic adenocarcinomas, finding *FZD5* (frizzled class receptor 5) as a common vulnerability that can be exploited therapeutically [50]. 

Despite the significant advantages that RNAi technology has given, several limitations should be considered: the appearance of unpredicted and uncontrolled siRNA off-target effects, lack of protein knockdown after mRNA silencing, low stability of siRNA oligonucleotides, and cell toxicity of transfection conditions [51]. The use of cancer cell lines in RNAi screenings represent a pillar, as they are easy to grow in standard culture conditions and have clonal origin giving rise to genetically homogeneous populations. However, over-passaged cell lines tend to accumulate additional mutations and to display a different phenotype, altering the biological readout. Normal cells, or primary cells derived from normal tissues, used as a control, grow in vitro for only a limited number of passages and must be immortalized; they therefore do not faithfully mimic the biological properties of an in vivo system. Another pitfall relies on the fact that the readout is usually short-term, allowing the detection of only strong phenotypes dealing with survival and proliferation. Therefore, many classes of genes, and consequently long-term effects, are neglected. Lastly, the use of individual cells in culture limits the study of genes that have a crucial role in sensing and perturbing the cellular microenvironment. All the limitations reported above can be overcome by the use of three-dimensional cultures, tissues, organoids, and even whole organisms.

In conclusion, targeting NOA genes represents a new treatment option, which can be pursued through (i) stress sensitization, which aims to switch off intracellular stress support pathways, or (ii) stress overload, which aims to induce the opposite effect. As two sides of the same coin, the consequence is the promotion of cell growth arrest and, finally, cell death.

## 2. Thyroid Cancer 

### 2.1. Molecular Alterations and Therapeutic Implications

TC accounts for 2.5% of all cancers and 90% of endocrine malignancies, with an incidence that has increased by 4.5% per year in the last decade. The majority of TCs originate from follicular cells and are grouped in the following different histotypes: papillary thyroid carcinoma (PTC) and follicular thyroid carcinoma (FTC), both defined as well-differentiated thyroid carcinoma (WDTC); poorly-differentiated thyroid carcinoma (PDTC); and anaplastic thyroid carcinoma (ATC). A small fraction of TC (~2%–5%), which originate from parafollicular C cells, are instead classified as medullary thyroid carcinomas (MTC) [52]. Several genetic and epigenetic alterations (i.e., rearrangements, somatic mutations, aberrant gene expression and methylation, micro RNA (miRNA) and long non-coding RNA (lncRNAs) deregulation) have been involved in the promotion of thyroid carcinogenesis. The main genetic lesions in TC consist of genetic rearrangements and point mutations. PTC are characterized by chromosomal rearrangements of *RET* [53] or *NTRK* (neurotrophic tyrosine kinase receptor) proto-oncogenes [54], whose activation is responsible for downstream signaling pathway that promotes cell growth, proliferation, and survival. A small fraction of PTC show gene rearrangements in *ALK* and *BRAF* genes, whereas rearrangements in *PPARγ* (peroxisome proliferator-activated receptor) gene are common in FTC [52]. The most frequent activating mutations in TC are the *V600E* mutation of *BRAF* gene and, less frequent, of the *RAS* (rat sarcoma) oncogene family (*N-RAS, K-RAS, H-RAS*), which are effectors of altered MAPK (mitogen activated protein kinase) and PI3K cascades. Even though in WDTC gene rearrangements and point mutations are almost mutually exclusive, PDTC and ATC display the co-presence of different lesions, including mutations in *TP53* (Tumor Protein P53*)*, *CTNNB1* (catenin beta 1), *IDH1* (isocitrate dehydrogenase (NADP)+) 1)*,* and *TERT* (telomerase reverse transcriptase) genes [52,55]. Two studies by the TCGA (The Cancer Genome Atlas) consortium have revolutionized the understanding of the genomic landscape of PTC, PDTC, and ATC [55,56]. A cohort of 496 PTC samples has been exhaustively analyzed by different approaches including next-generation DNA and RNA sequencing, copy-number analysis, DNA methylation, and proteomic assays. Besides the well-known driving oncogenic lesions in PTC, the study allowed the identification of novel driver mutations (i.e., *EIF1AX* (eukaryotic translation initiation factor 1A X-linked), *PPM1D* (protein phosphatase, Mg2+/Mn2+ dependent 1D)*,* and *CHEK2* (checkpoint kinase 2)) and gene fusions, and a better reclassification of tumors in molecular subtypes, useful to manage the disease from a clinical point of view. A similar approach was performed in PDTC and ATC [55]. Notably, functional studies demonstrated that *EIF1AX* mutations cooperate with *RAS* in inducing thyroid tumorigenesis and sensitize tumor cells to mTOR inhibition [57].

The TCGA study also contributed to the identification of different miRNA expression patterns, associated with differentiation and tumor progression, also useful to better define TC prognosis. miRNAs represent potential therapeutic targets [58], and functional studies support this issue, suggesting that fine-tuning of miRNA expression in thyroid cancer can modulate the activity of fundamental pathways. Several examples of the major miRNAs targeting proteins of the MAPK, PI3K, and TGFβ (transforming growth factor beta) pathways have been recently reviewed in [59].

lncRNAs are small RNA molecules that play an important role in different cell functions [60], and deregulation has been associated with TC initiation and/or progression [60,61] through their involvement in cellular functions such as promotion of proliferation, migration, invasion, metastasis, and pharmacological or radioiodine treatment resistance. Thus, some lncRNAs have been proposed as therapeutic targets [60]. Moreover, lncRNA detection in blood and cancer tissues may be helpful in diagnosis and prognosis, especially in PTC. 

Gene expression profile studies allowed the identification of other molecular mechanisms concerned with thyroid carcinogenesis, as well as genetic markers useful for diagnostic and prognostic purposes; the effect of their modulation in in vitro models allowed speculation that they may represent putative therapeutic targets. Genes overexpressed in thyroid tumors include, among others, *DUSP6* (dual specificity phosphatase 6) [62], *CITED1* (cbp/P300 interacting transactivator with Glu/Asp rich carboxy-terminal domain 1) [63], *S100A11* (S100 calcium binding protein A11) [64], and *TWIST1* (twist family BHLH transcription factor 1) [65]; their protumorigenic role has been assessed in preclinical models, showing that their inhibition reduces the thyroid tumor cell phenotype. Downregulation of gene expression in TC is often related to epigenetic mechanisms, such as aberrant DNA methylation [66], which accounts for the downregulation of tumor suppressor genes such as *RASSF1A* (ras association domain family member 1), *SLC5A8* (solute carrier family 5 member 8), *RARβ2* (retinoic acid receptor beta 2), and *RAP1GAP* (rap1 GTPase-activating protein 1)*,* as well as thyroid-specific genes, such as *TSHR* (thyroid stimulating hormone receptor) and *TPO* (thyroid peroxidase), and *SLC5A5* (solute carrier family 5 member 5) also known as *NIS* [67]. Several downregulated genes have been functionally classified as oncosuppressors, as their restoration attenuates the thyroid tumor cell phenotype. This is the case for *TIMP3* (tissue inhibitor of metalloproteinases 3) [68], as well as *IGFBP7* (insulin like growth factor binding protein 7) [69], *PTEN* (phosphatase and tensin homolog) [70], and *MT1G* (metallothionein 1G) [71]. 

### 2.2. Management of Thyroid Cancer Patients 

**Figure 3:**
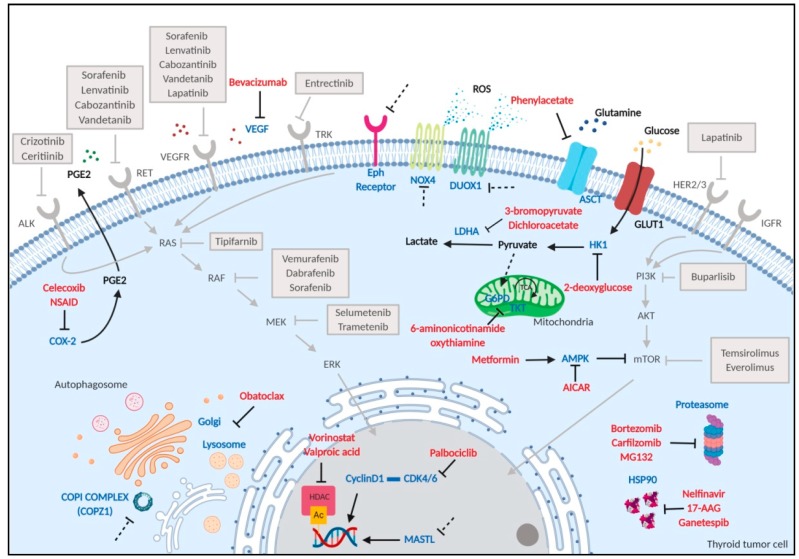
Scheme of therapeutic targets in thyroid tumor cells. Tyrosine kinase receptors, along with their downstream pathways, and pharmacological inhibitors entered in clinical practice are shown in gray; drugs, tested at pre-clinical and clinical level, targeting NOAs, are shown in red; the dotted lines represent putative NOAs whose inhibitor is not yet available. AICAR: 5-aminoimidazole-4-carboxamide-ribonucleoside; AKT: serine/threonine kinase 1; ALK: anaplastic lymphoma receptor tyrosine kinase; AMPK: adenosine monophosphate-activated protein kinase; ASCT: alanineserinecysteine-type amino acid transporter; CDK4/6: cyclin-dependent kinase 4/6; COPZ1: coatomer protein complex zeta 1; COX-2: cyclooxygenase-2; DUOX1: dual oxidase 1; Eph: ephrin; ERK: extracellular signal–regulated kinases; GLUT1: glucose transporter 1; G6PD: glucose-6-phosphate dehydrogenase; HDAC: histone deacetylase; HER2/3: erb-b2 receptor tyrosine kinase 2/3; HK1: hexokinase 1; HSP90: heat-shock protein 90; IGFR: insulin-like growth factor 1 receptor; LDHA: lactate dehydrogenase A; MASTL: microtubule associated serine/threonine kinase-like; MEK: mitogen-activated protein kinase; mTOR: mammalian target of rapamycin; NOX4: nicotinamide adenine dinucleotide phosphate NADPH oxidase 4; NSAID: nonsteroidal anti-inflammatory drug; PGE2: prostaglandine E2; PI3K: phosphatidylinositol-3-kinase; RAS: rat sarcoma viral oncogene homolog; RAF: rapidly accelerated fibrosarcoma; RET: rearranged during transfection; ROS: reactive oxygen species; TKT: transketolase; TRK: tropomyosin receptor kinase; VEGF: vascular endothelial growth factor; VEGFR: vascular endothelial growth factor receptor. Figure created with BioRender.

The majority of patients with WDTC are effectively treated, with a 10-year survival rate over 90%. Standard therapy approaches include the surgical removal of the thyroid gland, thyroid-stimulating hormone (TSH) suppression, and radioactive iodine 131 (RAI) treatment for residual thyroid tissue ablation [72]. Nevertheless, local recurrence occurs in up to 20% of patients and distant metastasis in approximately 10% by 10 years [73], and metastatic PTC and FTC often become refractory to radioactive iodine therapy [74]. ATC are generally resistant to classical therapies and responsible for more than half of all TC-related mortalities [72]; thyroidectomy is not always feasible due to the local extension of the disease and invasion of contiguous anatomic structures. To date, RAI refractory TC and ATC represent the most important clinical problem, for which new therapeutic options are needed. To face this need, molecular findings in thyroid cancer have been translated into clinics. The thyroid-driving genetic alterations described above involve genes acting through the MAPK and PI3K/AKT pathways; on this premise, various components of this signaling cascade have been explored as therapeutic targets. The block of these intracellular signaling cascades through the use of tyrosine kinase inhibitors (TKIs) has been explored; several compounds have been developed and approved for treatment of patients with advanced TC who are inoperable, show evidence of progression disease, and exhibit RAI resistance. A complete description of molecules available in clinical practice has been exhaustively reviewed in [9,52] and are reported in Figure 3. However, even if multiple kinase inhibitors have had some clinical benefit, their association with improvements in overall survival is still questionable. Other therapeutic approaches consist of the use of demethylating and re-differentiating agents, inhibition of proteosome and histone deacetylases, and immunotherapy, which, to date, represents the most promising treatment option for advanced thyroid diseases [52,75].

In conclusion, even though much progress has been made in the treatment of thyroid cancer thanks to the development of a variety of molecular-targeted agents, new effective therapeutic options are still needed.

## 3. Discovery of Vulnerabilities in Thyroid Cancer: Our Experience

### 3.1. Functional Screening

As anticipated in the first paragraph, the identification of tumor cell vulnerabilities is achieved by loss of function RNA interference-based genetic screenings. The NOA concept is completely novel in the context of thyroid cancer; nevertheless, it may represent a powerful tool for identification of novel therapeutic approaches for the most aggressive and advanced cases that are not cured by standard therapy. 

To identify genes selectively promoting lethality of thyroid tumor cell lines, we, at first, exploited a screening of synthetic siRNA library targeting 9031 human genes using the BCPAP tumor cell line (carrying the *BRAFV600E* mutation) and immortalized normal human thyrocytes (N-Thy-ori3-1) as a control [76]. The effect of each siRNA on cell viability was analyzed by a medium-high throughput colony formation assay (following the same in vitro approach reported in Figure 2a). Comparison of data from the two cell models identified vulnerabilities common to BCPAP and N-Thy-ori3-1 (270 genes, considered lethal hits, encoding proteins involved in survival, proliferation, and proteosome machinery), as well as specific for BCPAP cells (386 genes, considered differential hits). Further confirmatory studies generated a panel of 15 putative differential hits involved in cell cycle control, vesicular transport, glucose and fatty acid metabolism, and intracellular signaling transduction. Notably, the list included BRAF, in keeping with the known dependency of BCPAP cells on BRAFV600E oncogene activity. The mutational status and expression of hit genes in PTC and normal thyroid was assessed in the thyroid TCGA dataset. Except for the expected high frequency of oncogenic *BRAF* mutations, no mutations affecting the other genes were detected, suggesting the absence of functional alterations in PTC. A group of hit genes (*CCND1* (cyclin D1), *RGS3* (regulator of G protein signaling 3), *OXTR* (oxytocin receptor), *RASD1* (ras related dexamethasone induced 1), *DNM3* (dynamin 3)) showed overexpression in PTC with respect to normal thyroid; a second group (*COPE* (coatomer protein complex subunit epsilon), *COPZ1* (coatomer protein complex subunit zeta 1), *PLA2G15* (phospholipase A2 group XV), *SRPK1* (serine/arginine-rich protein-specific kinase 1), *REM2* (RRAD and GEM like GTPase 2), *EPHB4* (ephrin type-B receptor 4), *BRAF*) displayed equal or slightly different expression; a third group (*MAP4K5* (mitogen-activated protein kinase kinase kinase kinase 5), *NUDT9* (nudix hydrolase 9), *MASTL* (microtubule associated serine/threonine kinase-like)) resulted in down-regulated expression. Validation as vulnerability for thyroid cancer has been performed for three hits (see Section 3.2), whereas for the majority of genes it remains to be investigated.

A similar approach was pursued by Cantisani et al. who, using a siRNA library targeting the human kinome and related proteins, identified genes belonging to the EPH (ephirin) receptor tyrosine kinase, SRC (SRC proto-oncogene, non-receptor tyrosine kinase), and MAPK (mitogen activated protein kinase) families as necessary for the viability of thyroid tumor cells, but not for normal cells, and proposed them as potential novel therapeutic targets for thyroid tumors [77]. Further validation remains to be performed.

In summary, functional screening allowed the identification of several vulnerabilities for thyroid cancer. Even though not all fall strictly in the NOA definition (i.e., are not mutated, but some show variations in expression in tumor with respect to normal thyroid), they nonetheless represent an Achilles’ heel that could be targeted.

### 3.2. Validation of NOA Targets 

Cyclin D1, encoded by the *CCND1* gene, promotes the G1/S phase transition through the activation of Cdk4 and Cdk6 kinases (CDK4/6) [78]. *CCND1* deregulation in tumors is frequent as consequence of gene mutations, amplifications, or protein overexpression. Cyclin D1 overexpression is mainly due to oncogenic signaling through the RTK and MEK (mitogen-activated protein kinase)-ERK (extracellular signal–regulated kinase) pathways, as well as translocation and amplification of the *CCND1* gene; moreover, it may also result from deregulation of mRNA stabilization, nuclear export and ubiquitin-mediated proteolysis, or presence of the D1b splicing variant [78]. Cyclin D1 has been proposed as a cancer therapeutic target; however, its direct targeting is difficult, as it lacks enzymatic activity. Approaches based on the inhibition of CDK4/6 have been developed; after promising results in preclinical studies and clinical testing, three CDK inhibitors, palbociclib, ribociclib and abemaciclib, have recently received FDA (Food and Drug Administration) approval for hormone receptor positive metastatic breast cancer [79]. In TC, cyclin D1 overexpression at the mRNA and protein levels has been documented, and proposed to contribute to tumor progression [80,81,82]. Moreover, *CCND1* gene polymorphic variants in patients with WDTC have been identified [83], and the splice variant cyclin D1b has been proposed as a novel diagnostic and predictive biomarker for TC [84]. Recently, *CCND1* has been identified as a key gene by integrated bioinformatics analysis [85]. Nevertheless, the role of *CCND1* in thyroid tumorigenesis has been poorly investigated, except for recent papers showing *CCND1* as a direct target of deregulated miRNAs and long non-coding RNAs [86,87,88]. We identified cyclin D1 as vulnerability for TC cells, as its siRNA-mediated inhibition causes consistent growth reduction of different thyroid tumor cell lines, but not normal ones. Moreover, the treatment of thyroid cancer cells with the CDK4/6 inhibitor palbociclib (PD-0332991) induces a consistent antiproliferative effect [76]. In keeping with our results, other groups have confirmed the efficacy of palbociclib in in vitro and in vivo preclinical models of TC, also in combination with a PI3K/mTOR inhibitor to bypass mechanisms of therapy resistance [89,90,91]. On the whole, we are confident that the evidence that cyclin D1 represents TC cell vulnerability may prompt preclinical and clinical studies employing CDK4/6 inhibitors for treatment of TC.

MASTL (microtubule associated serine/threonine kinase-like) is involved in mitosis regulation; it inhibits the PP2A/B55δ protein phosphatase complex responsible for dephosphorylation of CDK1 substrates. MASTL activity is necessary for chromosomal segregation and to prevent prometaphase arrest and mitotic failure [92]. Furthermore, it suppresses the DDR and promotes checkpoint recovery and cell cycle progression [93,94]. The role of MASTL in regulating mitosis and involvement in cancer has been extensively reviewed in [95]. MASTL is overexpressed in prostate, head and neck, colon, and breast cancer [95,96,97,98]. In breast cancer, MASTL overexpression is associated with patient poor prognosis [99], more advanced clinical stage, [96] and reduced survival [100]. A recent study revealed that MASTL promotes proliferation and mitotic entry of human liver cancer cells upon induction of proinflammatory cytokines (i.e., IL-6, TNF (tumor necrosis factor)-alpha) [98]. At variance with other tumor types, TC shows neither overexpression nor mutations of *MASTL* [76,101]. Our functional studies identified *MASTL* as a vulnerability gene for TC, being essential for cell growth. Moreover, its depletion (i) increases the percentage of cells presenting nuclear anomalies and aberrant mitotic figures, which are indicative of mitotic catastrophe; (ii) enhances DNA damage, observed as the presence of increased γH2AX (H2A histone family member X) foci number; (iii) sensitizes thyroid tumor cells to cisplatin; and (iv) induces apoptotic cell death. As a consequence of the high number of mutations that alter mitosis fidelity and cause chromosome mis-segregation, tumor cells undergo elevated mitotic stress, thus becoming dependent on stress support pathways [102]. On this basis, our results strongly support the notion that MASTL could be considered as non-oncogene in the context of thyroid tumors, exerting a central role in attenuating mitotic, replication, and DNA damage stress. As for other tumor types, both sensitization and overload of mitotic stress have been exploited as therapeutic for TC; targeting mitotic machinery elements, such as PLK1 and Aurora kinases through small-molecule inhibitors, significantly reduced growth and induced cell death in ATC-derived cell lines [103,104]. MASTL has been proposed as a novel therapeutic target in different tumor contexts; more interestingly, its inhibition sensitizes squamous cancer cells to cisplatin [96], colon cancer cells to 5-fluorouracil [105], and lung and breast cancer cells to radiation therapy [106,107]. To date, many efforts are being made to find new therapies based on MASTL targeting. The evidence that MASTL deletion is not deleterious in conditional knockout adult mice [108] strengthens the potentiality of this approach. Ocasio et al. designed the “first generation inhibitor” that is capable of inhibiting MASTL kinase activity [109], and Ammarah et al., after screening a library of natural and synthetic compounds, identified the ZINC53845290 compound as the most promising MASTL inhibitor [110]. 

COPZ1 (coatomer protein complex zeta 1) is a member of the COPI (coatomer protein complex I), exerting a role in vesicular trafficking from the Golgi apparatus to the ER, as well as endosome maturation, autophagy [111,112], viral infection [113], and lipid homeostasis [114]. Few investigations have dealt with the role of COPZ1 in cancer. Shtutman at al. attributed to COPZ1 an essential role in different tumor types (i.e., prostate, breast, and ovarian carcinoma), indeed demonstrating that depletion induces Golgi apparatus collapse, autophagy inhibition, and apoptotic cell death in proliferating and dormant cells [115]. Interestingly, the same authors found that COPZ1 vulnerability of tumor cells relies on the downregulation of the isoform COPZ2, whose gene hosts the well-known oncosuppressor miRNA152 [115,116]. We found that COPZ1 represents vulnerability for TC, as it is required for the viability of different TC cell lines but not of normal thyrocytes [117]. Moreover, the absence of mutation and aberrant expression in PTC [76] renders COPZ1 a good example of NOA for thyroid cancer cells. COPZ1 depletion causes abortive autophagy, ER stress, unfolded protein response (UPR), and apoptosis, suggesting a central role in coping with cellular proteotoxic stress. In addition, local treatment with siRNA oligos targeting COPZ1 reduces tumor growth of TC xenograft models [117]. Notably, there is currently a strong interest in developing selective small-molecules targeting the coatomer complex, which may be useful to treat not only TC, but also different types of cancers. Interestingly, members of the complex have been recently proposed as therapeutic targets in cancer such as COPA (coatomer protein complex subunit alpha) for mesothelioma, and COPB2 (coatomer protein complex subunit beta 2) for colorectal cancer, lung adenocarcinoma, cholangiocellular carcinoma, and gastric cancer [118,119,120,121,122,123]. Other strategies to impair the ER–Golgi network are being currently pursued as anti-cancer therapy. For example, the M-COPA (2-methylcoprophilinamide) compound, inhibiting Arf1 (ADP ribosylation factor 1) involved in COPI and clathrin-coated transport vesicles, has been shown to disrupt the Golgi apparatus and RTK translocation to the membrane, to have antitumor effects against MET-addicted gastric cancers, and to overcome TKI resistance in EGFR-mutated non-small-cell lung carcinoma [124,125].

## 4. Other Putative NOAs

In the context of thyroid tumors, several functional studies on pathway interactions and their signaling have opened up new routes for therapeutic targets. Many have allowed the identification of NOAs, belonging to hallmarks of cancer reported in Section 1, which have been preclinically investigated and may be considered as effective in treating TC (Figure 3). Hereafter, some examples of less-explored TC vulnerabilities are reported.

Physiologically, thyrocytes produce substantial amounts of ROS for synthesis of T3 and T4 hormones, and thus the thyroid gland displays an “oxidative nature” that, when overloaded, can initiate genomic instability and malignant transformation [126]. Ionizing radiation, but also the activity of the RAS and BRAF oncogenes, up-regulates the expression of DUOX1 (dual oxidase 1) and NOX4 (nicotinamide adenine dinucleotide phosphate NADPH oxidase 4), two oxidases responsible for generation of ROS [126,127], which can be considered as examples of non-oncogenes to be targeted. Inhibitors for DUOX1 and NOX4 have been identified [128], but not yet tested in thyroid tumors. On the other hand, applying oxidative stress overload can specifically kill thyroid cells. For example, the inhibition of the pentose phosphate pathway through inhibitors of G6PD (glucose-6-phosphate dehydrogenase) and transketolase (6-aminonicotinamide and oxythiamine, respectively increase production of ROS, and induce ER stress and TC cell death [129].

To date, the use of epigenetic drugs, specifically HDAC (histone deacetylase) inhibitors such as vorinostat and valproic acid, has been widely studied in thyroid cancers. This approach, essentially causing the arrest of tumor growth, differentiation, apoptosis, and sensitization to radiation, showed the most efficacy in combination with chemotherapy [130,131] and with HSP and proteosome inhibitors [132]. Although none of the HDAC inhibitors have produced significant responses in clinic studies to date, further research in the field of HDAC inhibitors and their functions, other than DNA deacetylation, such as buffering proteotoxic stress, should be performed. Interestingly, it has been demonstrated that inhibiting HDAC is a strategy to reactivate MHC (major histocompatibility complex) gene expression in tumor cells, enhancing tumor immune surveillance [133]. All the above considerations corroborate the idea that HDACs deserve to be classified as NOAs in TC.

Similar to other tumor types, TCs display sensitivity to proteasome inhibition. Compounds such as bortezomib, MG132, and carfilzomib have shown efficacy in killing cells in preclinical thyroid cancer models [134,135,136]. 

The HIV (human immunodeficiency virus) protease inhibitor nelfinavir (NFV) is an example of drug repositioning, as recently it has been introduced as a therapeutic option for the treatment of pancreatic cancer, non-small-cell lung cancer, liposarcoma, and glioblastoma multiforme [26]. It has been demonstrated that NFV acts as an inhibitor of HSP90 and causes unfolded protein response and ER stress [137]. Moreover, it synergizes with proteosome inhibitors to suppress cancer cells [138,139,140]. Interestingly, a recent study demonstrated that as NFV inhibits thyroid cancer cell proliferation and migration, it also induces DNA damage and sensitizes cells to anoikis, suggesting that it could be a new promising therapeutic agent for TC [26,141]. Several other HSP90 inhibitors, such as 17-allylamino-17-demethoxygeldanamycin (17-AAG) and ganetespib have been tested in preclinical models with the aim to cope with intracellular proteotoxic stress. Their cytotoxicity has also been demonstrated in thyroid tumor cells [142,143], although its clinic effectiveness has not yet been assessed.

TC undergoes metabolic adaptations that could be used as therapeutic targets. The majority of metabolism-related molecules, such as GLUT1 (glucose transporter 1), HK1 (hexokinase 1), LDH-A (lactate dehydrogenase A), GLS1 (glutaminase), and MCT1 (monocarboxylate transporter 1) are upregulated in different subtypes of thyroid carcinoma [144]. Inhibitors of glutamine uptake (phenylacetate), glucose metabolism (2-deoxyglucose, 3-bromopyruvate, dichloroacetate), and activators of adenosine monophosphate-activated protein kinase (AMPK; metformin and 5-aminoimidazole-4-carboxamide-ribonucleoside) have all been proposed to regulate metabolic alterations in TC [145].

Champa and co-authors found that poorly differentiated and ATC cell lines, generally known to be intrinsically resistant to cell death, showed reduced tumor growth both in vitro and in vivo when treated with obatoclax, a pan-inhibitor of the anti-apoptotic proteins of the BCL2 (BCL2 apoptosis regulator) family. Interestingly, they demonstrated that obatoclax induced necrosis in cancer cells, but not in non-transformed thyroid cells, through the destabilization of lysosomes. The same effect was observed using other lysosome-targeting compounds, such as mefloquine and LLOMe (L-leucyl-L-leucine methyl ester), suggesting that impairment of lysosomal machinery can be considered an Achilles’ heel of advanced TC cells [146]. Other compounds such the anti-malaria drug chloroquine, which blocks autophagy by inhibiting lysosomal proteases and autophagosome–lysosomal fusion, has been considered as an example of repositioning therapy for TC [26].

Cycloxygenase-2 (COX-2), an important enzyme involved in the synthesis of prostaglandins and inflammation, has been proposed as a NOA for different tumor types, as it is responsible for progression of tumors, resistance to cell death, metastasis, and for onset of immunosuppressive milieu [13,31]. In the context of TC, the COX-2/PGE2 (prostaglandine E2) pathway has been demonstrated to exert a role in proliferation and invasion of thyroid tumor cells and to be associated with tumor recurrence in WDTC [147]. Moreover, Park et al. found that TC cells, through the secretion of PGE2, are able to evade immune surveillance by mitigating the cytolytic activity of natural killer cells in the tumor microenvironment [148]. However, administration of celecoxib, a COX-2 inhibitor, in thyroid cancer patients did not give encouraging results due to the lack of efficacy and high toxicity [131,149], suggesting that other compounds should be tested for their therapeutic benefits.

## 5. Conclusions

Despite the huge efforts in dissecting the molecular alterations in TC, which have nevertheless allowed the introduction of several therapeutic options into clinical practice, the management of patients with aggressive and iodine-refractory thyroid tumors is still challenging. Thus, there is a need to identify new strategies for treatment. Several lines of evidence have shown that non-oncogene addictions can be considered as new therapeutic options. As for other tumors, in the context of thyroid tumors it is very difficult to exactly define into which category some genes and pathways should fall—oncogenes or non-oncogenes? The reasons can be varied. Studies performed in some tumor types cannot be translated to the thyroid, as cell specificity is a determining factor for establishing whether a gene acts as oncogene or non-oncogene. Many functional studies neglect the study of the normal counterpart, and therefore do not highlight differential lethality in normal and tumor cells. As reported above, one of the biggest pitfalls is the limited availability of normal cell models, and the thyroid is no exception. Moreover, the majority of vulnerabilities discovered deal with proliferation and survival, and the pillar functions involved in the tumor microenvironment are lacking. Despite the efforts being made in the discovery of new targets, NOAs included, there is still an evident difficulty to enter novel drugs into clinical trials to assess clinical validation and, consequently, patient benefit.

We are aware that this review is not fully exhaustive, as many NOAs in the context of thyroid tumors, especially those belonging to DNA damage, replication, and mitotic stress, have not been expanded upon. Considering the different functional categories in which NOAs fall and large amount of information available in literature, many concepts have been consequently omitted.

In conclusion, there is robust evidence that NOAs represent novel molecules that should be tested at the preclinical and clinical levels. Their targeting, alone or in association with standard therapy, could offer new and advantageous therapeutic approaches for treatment of thyroid cancers.
